# ADHD-related attentional variability and its impact on postural stability in young adults

**DOI:** 10.3389/fnhum.2026.1765636

**Published:** 2026-02-05

**Authors:** Çiğdem Yazici-Mutlu, Can Seçinti, Aber Ahmetoğlu

**Affiliations:** Department of Physiotherapy and Rehabilitation, Health Science Institution, Yeditepe University, Istanbul, Türkiye

**Keywords:** ADHD, attention, motor behavior, postural stability, sensory integration

## Abstract

**Purpose:**

Postural control is influenced by attentional demands, yet the role of attention-related individual differences in young adults remains underexplored. This study examined whether sustained attention performance is associated with postural stability in individuals with and without potential ADHD traits (pADHDt)-related attentional variability.

**Methods:**

Forty-two adults (18–35 years) were grouped based on ASRS scores into a pADHDt group (ASRS > 14; *n* = 21) and a comparison group (ASRS < 14; *n* = 21). Sustained attention was assessed using the Sustained Attention to Response Task (SART). Postural stability was measured with the ProKin 252 stabilometer across three stance conditions (two-foot, tandem, soft surface), with and without a verbal cognitive task (backward counting), under eyes-open and eyes-closed conditions. Center of pressure (CoP) sway area and medial–lateral (ML) and anterior–posterior (AP) sway velocities were analyzed using mixed-design ANOVA with Bonferroni correction.

**Results:**

Participants in the pADHDt group made more SART errors and showed slower reaction times than those in the comparison group (*p* < 0.05). During cognitively demanding standing tasks, the pADHDt group exhibited larger CoP sway areas in two-foot and tandem stances with eyes open, and in tandem stance with eyes closed (*p* < 0.05). No group differences were observed in ML or AP sway velocities.

**Conclusion:**

Adults with ADHD-related attentional variability exhibit differences postural stability under increased cognitive demands, suggesting a role of attentional control in balance-related motor behavior in young adults with pADHDt.

## 1 Introduction

Attention Deficit Hyperactivity Disorder (ADHD) is classified as disorders usually diagnosed in infancy, childhood, and adolescence in the *Diagnostic and Statistical Manual of Mental Disorders* (DSM-IV) (American Psychiatric Association, 1994). ADHD was then seen as a childhood disorder and, in the fourth edition of the DSM, published in 1994. The diagnostic criteria for ADHD have been revised in the DSM-5 and are defined as Neurodevelopmental Disorders. As a condition that primarily affects attention regulation, impulse control, and executive functioning, ADHD manifests in various cognitive and behavioral difficulties especially during childhood and adolescence. In children, it commonly presents with impairments in both motor and inhibitory control. Motor deficiencies are observed in approximately 30–50% of cases (Goulardins et al., 2017; Lachambre et al., 2021). Despite not being included in the diagnostic criteria, motor control problems are prevalent in children with ADHD and affect up to 50% of them. Research has consistently shown that children with ADHD struggle to maintain balance (Pitcher et al., 2003). Zang and Qian, among others, reported significant balance impairments in children with ADHD, particularly during challenging conditions such as standing on a foam surface with eyes open or closed. Children with ADHD experience balance deficits in simple standing postures and exhibit more pronounced deficits in challenging standing conditions (Zang and Qian, 2015). Furthermore, Bucci et al. (2014) reported that children with ADHD demonstrate increased postural sway and poorer visual fixation performance. The literature also highlights developmental and gender-related differences in postural control, with males typically exhibiting greater instability than females. In line with these findings, Ren et al. (2014) reported that boys with ADHD exhibit poorer static postural control compared to typically developing peers.

ADHD, traditionally associated with childhood, is now recognized in adults as well, with a global prevalence of 3–7% (Song et al., 2021). A meta-analysis showed that while 15% retain a full diagnosis into adulthood, 50% show partial remission (Faraone et al., 2006). Of late years, there is growing awareness that ADHD symptoms, like inattention, can persist into adulthood (De Graaf et al., 2008). In adult ADHD, with it is features of attention deficits, impulse control issues, and activity level problems, can have adverse effects on an individual’s work capacity and performance (Edvinsson et al., 2017).

While posture refers to the static position of the body, postural control is a complex process based on the coordinated operation of various physiological and neurological systems (Brooks, 1986). The postural control system relies on sensory input from the visual, somatosensory and vestibular systems to perform motor actions and reorganize among themselves each time according to the situation (Horak, 2006). The contributions of the senses are constantly changing and reorganized each time. Early theories in this area suggested that postural responses arise from central set adjustments involving cognitive and neuromotor state changes based on basic sensorimotor inputs; this idea was later confirmed by evidence implicating cortical modulation in refining these responses through central set regulation (Jacobs and Horak, 2007; Prochazka, 1989). The negative effects of reduced attention on the achievement of conscious postural control, stability and movement coordination are emphasized by two theories. One of these theories is the Reinvestment Theory (RT). This theory focuses on the fact that conscious attention will impair postural control (Seidler et al., 2010), while the other theory, the Constrained Action Hypothesis (CAH), is related to the direction of attentional focus and suggests that external focus improves performance during dual tasks by enabling automatic motor function (Wulf et al., 2001). The dual-task and effect on the postural performance have been reported in children and adolescence with ADHD (Alsamiri, 2025; Caldani et al., 2023; Leitner et al., 2007).

Although studies have been conducted with children and adolescents with ADHD, it is unclear whether balance problems persist into adulthood. There are very few studies which have addressed disparities in postural control in adults with ADHD compared to adults without ADHD (Hove et al., 2015; Jansen et al., 2019). One of the key challenges in this area is the heterogeneity of individuals presenting with ADHD traits, which are now widely understood as existing on a spectrum. In the present study, we intentionally focused on a non-clinical sample of individuals who exhibited elevated ADHD traits based on the ASRS-5, yet had no formal diagnosis of ADHD. Moreover, investigating attentional performance in non-diagnosed individuals contributes to a better understanding of cognitive variability in the general population and may inform early identification or preventive strategies in at-risk groups.

Based on these gaps in the literature, the aim of this study was to compare adults with potential ADHD traits (pADHDt) and healthy control in terms of postural stability and their ability to direct and sustain attention during dual postural tasks and changing difficulty balance tasks performance. Demonstrating the effect of attention on postural control in adults with ADHD is essential in establishing balance training protocols. Because of all this information, the study aims to explore the connection between postural control and sustained attention in individuals with the pADHDt.

## 2 Method

### 2.1 Subjects

This study included 42 healthy young adults (18–35 years). *An a priori power analysis was conducted using G*Power 3.1 for a mixed-design ANOVA (within–between interaction). Assuming a small-to-moderate effect size (*f* = 0.20), an alpha level of 0.05, and a desired power of 0.80, the analysis indicated that a total sample size of 36 participants. While power analysis suggested a minimum of 36 participants, we recruited 42 (21 per group) to reduce the risk of dropout and to preserve group balance. All participants completed the study. The study ethical approval was obtained from the Marmara University Ethics Committee (no: 25.01.2024/02) and conducted between February and May 2024 at the Yeditepe University Physiotherapy and Rehabilitation Department.

Individuals with balance problems, recent injuries, neurological conditions, cognitive-affecting medications, ankle sprain history, or regular exercise in the past year were excluded. After obtaining informed consent, participants completed the Adult ADHD Self-Report Scale (ASRS-5), aligned with DSM-5 criteria. The ASRS-5 was not used as a diagnostic inclusion criterion but as a trait-based screening tool for group allocation. A cut-off score of ≥14 was applied to classify participants with higher ADHD-related attentional traits, while those scoring below this threshold were classified as having lower attentional traits. No objective cognitive performance test was used as an inclusion or exclusion criterion, as such tests reflect state-dependent task performance rather than stable attentional characteristics (Ustun et al., 2017).

Based on ASRS-5 scores, participants were assigned to two equal groups: Group 1 (pADHDt; group ASRS >14, *n* = 21) and Group 2 (Control group; ASRS ≤14, *n* = 21).

At baseline, all participants from both groups interviewed to collect their demographic information such as, age, height (cm), weight (kg), body mass index (BMI), gender (female/male), and physical activity level. Then, they completed SART exam measuring cognitive abilities. In addition, a force plate was used to evaluate the participants’ ability to maintain their balance in different physical and cognitive conditions.

### 2.2 Evaluation instruments

#### 2.2.1 Adult ADHD screening scale (ASRS-5)

The ASRS-5 is an ADHD screening tool aligned with the DSM-5 diagnostic criteria, introdused by the WHO. This scale is a 5-point Likert-type scale, consisting of 6 items, and underwent a validity study conducted by Üstün et al., which demonstrated strong psychometric properties (Ustun et al., 2017). The original ASRS-5 exhibited a sensitivity of 91.4%, a specificity of 96%, and an area under the curve (AUC) value of 0.94. Correlations with clinical diagnoses were established in both population and clinical samples. Genç et al. (2021), translated and tested the Turkish version, confirming its validity and reliability. A cut-off score of ≥14 on the ASRS-5 was used to identify individuals with elevated ADHD-related attentional traits, consistent with prior validation work proposing this threshold for screening purposes in adult populations (Ustun et al., 2017). Participants individually respond to the 6 questions, assigning scores in a 0–4 range, resulting in a total score ranging from 0–24. A score of 14 or higher suggests the possibility of ADHD.

ASRS-5 based upon the frequency of the following 6 item:

- How often do you have difficulty concentrating on what people say to you, even when they are speaking to you directly?
- How often do you leave your seat in meetings or other situations in which you are expected to remain seated?
- How often do you have difficulty unwinding and relaxing when you have time for yourself?
- When you are in a conversation, how often do you find yourself finishing the sentences of the people you are talking to before they can finish them themselves?
- How often do you put things off until the last minute?
- How often do you depend on others to keep your life in order and attend to details?

#### 2.2.2 Cognitive test: the sustained attention to response task (SART)

The SART is a 5-min computer-based test used to assess sustained attention designed by Robertson et al. (1997).

Participants were shown digits from 0 to 9, one at a time. They pressed the spacebar for every number except 3, which required no response. Each number appeared for 250 ms, followed by a 900 ms mask, with a total interval of 1,150 ms between digits. To make the task more challenging, digits were randomly shown in five different font sizes. Accuracy and speed were both emphasized. The test was completed on the PsyToolkit platform.

Before the test, participants received instructions and completed a brief practice trial of the SART. During the task, they pressed the spacebar for all digits except ‘3’, which appeared randomly and required no response. The test lasted approximately five minutes and included 225 trials. Participants responded using their dominant hand. Upon completion, the number of errors and mean reaction time (RT) were displayed. We also analyzed a correct response (Go correct RT) and for a wrong response (No-Go RT).

#### 2.2.3 Assessment of postural control

Participants were assessed using the ProKin 252 stabilometric device to assess participants sways of the CoP during standing. It is a proprioceptive system used for static and dynamic balance assessment and training. The device shows multiple programs for balance assessment and treatment. For this experiment, the “Static Stability Assessment Program” was chosen to provide detailed and precise data of each participant while static standing through the stabilometry platform. Stabilometry allows participants to be evaluated by detecting the sway of the CoP during static standing. Postural sway parameters: Average Antero-Posterior (A-P) Sway Velocity (mm/s), Average Medium-Lateral (M-L) Sway Velocity (mm/s) and Ellipse Area [total sway area, which is circumscribed by the COP (mm2)]. That data were obtained directly from the device software which provided preprocessed output files for each trial. No additional signal filtering or processing was applied. All values were exported in standard metric units and used as provided for statistical analysis.

**Figure 1:**
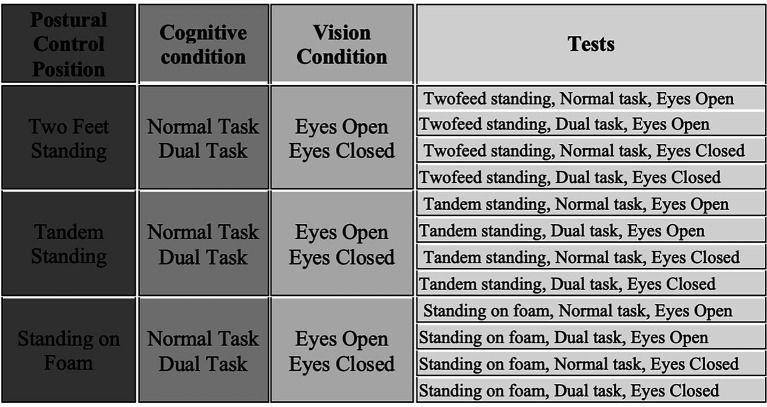
Schematic representation of the assessment under three stance conditions.

The assessment was conducted under three stance conditions: two-foot, tandem (heel-to-toe), and foam surface. Each was tested with eyes open and eyes closed, and under single-task (no cognitive task) and dual-task (counting backward from 100 by 3) conditions. The dual-task involved counting backward from 100 by threes, a procedure commonly used in previous research to induce cognitive load during postural control assessments (Fletcher and Osler, 2021; Granacher et al., 2011) (Figure 1).

**Figure 2:**
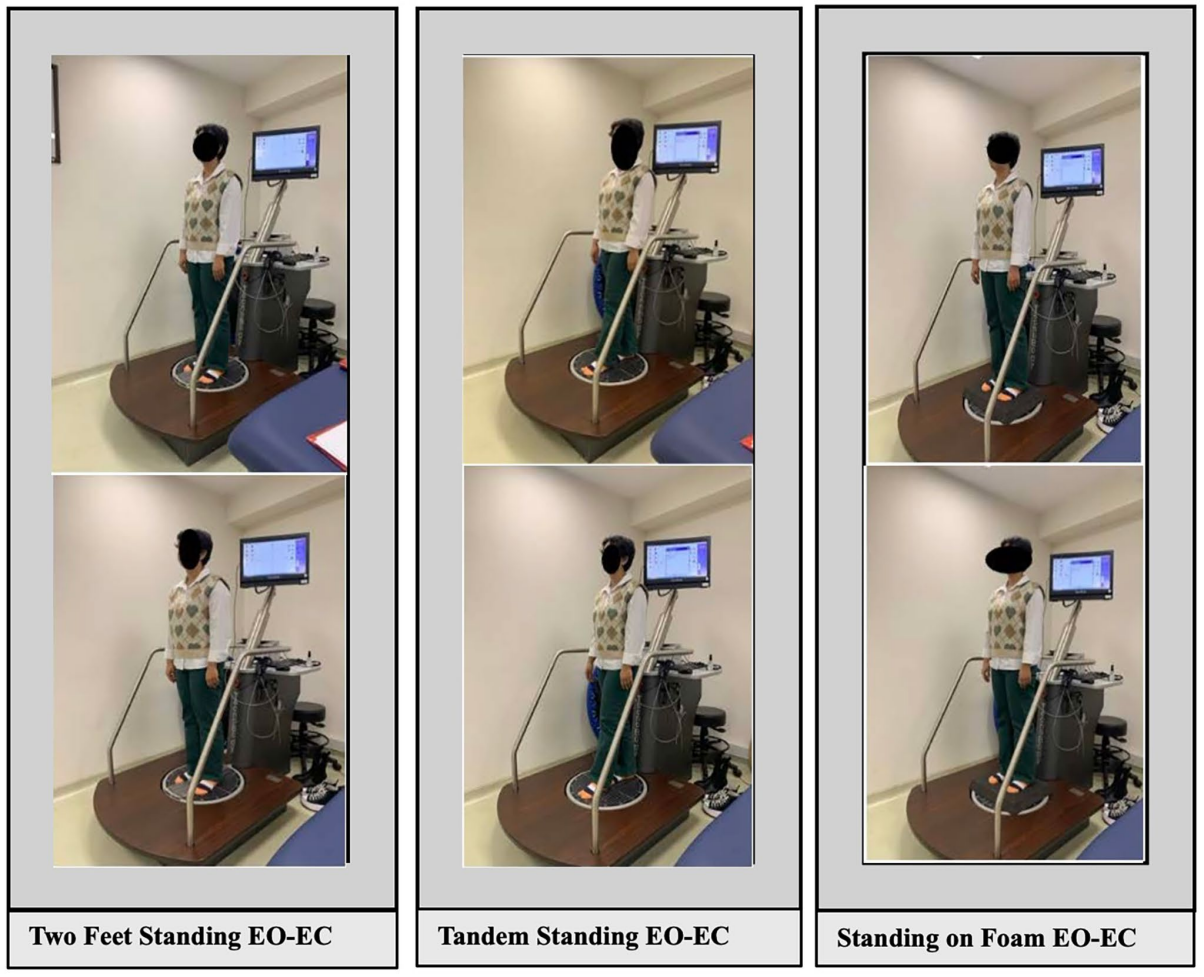
Illustration of the assessment procedure under three stance conditions.

The assessment procedure was carried out by following the steps below:

- After the procedure was explained, participants were instructed to stand barefoot on the platform facing away from the screen, aligning their feet precisely to the reference points with a 10 cm distance between them and an angle of 15–30° from the medial to lateral direction. Arms were positioned relaxed at the sides, the head was held in a neutral forward-facing position, and participants were asked to maintain this posture while fixating on a designated point for 30 s during each trial.
- For the tandem position; participants placed their feet in front of each other on the center line of the platform (with the dominant foot in front), facing a marked point on the wall. They maintained the same position with eyes open and eyes closed.
- For standing on the foam surface; a foam mat covering the entire surface of the power plate was placed under the participants’ feet. Participants were asked to stand upright on this foam surface.
- All postural control assessments were performed with/without dual-task and eyes open/eyes closed.
- In the eyes-open condition, participants were instructed to fixate on a marked point on the wall, while in the eyes-closed condition, the same foot position was maintained, but participants simply closed their eyes.
- The dual-task process is achieved by asking the participant to count backward from 100 to 3.
- Participants stood barefoot in specific positions for 30 s per trial, repeated three times per condition. All tests were done in random order, with 1-min rests in between. In total, the setup allowed evaluation of how different visual, stance, and cognitive loads affect balance (Figure 2).

### 2.3 Statistic analysis

Data were analyzed using IBM SPSS Statistics Standard Concurrent User Version 26 (IBM Corp., Armonk, NY, United States). Descriptive statistics were presented as mean ± standard deviation for normally distributed variables and as median with interquartile range for non-normally distributed variables. Categorical variables were expressed as frequencies and percentages.

Normality was assessed using skewness and kurtosis values, with absolute skewness values < ±2.0 and kurtosis values < 7.0 considered indicative of normal distribution (Kim, 2013). All outcome variables met these criteria.

Baseline demographic characteristics were compared between groups using independent-samples t-tests for continuous variables and chi-square tests (Pearson chi-square or Fisher’s exact test) for categorical variables.

To examine postural control outcomes, a 2 × 3 × 2 × 2 mixed-design analysis of variance (ANOVA) was conducted with Group (pADHDt vs. Control) as the between-subjects factor, and Posture/Surface (bipedal stance, tandem stance, foam surface), Vision (eyes open, eyes closed), and Task (single-task, dual-task) as within-subjects factors.

Main effects and interaction effects were tested. When the assumption of sphericity was violated, Greenhouse–Geisser corrections were applied. Significant main effects or interactions were followed by Bonferroni-corrected pairwise comparisons to identify specific differences between conditions.

Effect sizes were reported using partial eta squared (η^2^ₚ), with values of approximately 0.01 considered small, 0.06 medium, and 0.14 or greater large effects. Statistical significance was set at *p* < 0.05.

## 3 Results

Group 1 (pADHDt group) had a median age of 22 years, whereas group 2 (control group) had a median age of 24 years. Age, gender, body mass index, sensory sensitivity, and physical activity characteristics were similar in the study groups (*p* > 0.05) (Table 1). In the present study, the ASRS-5 cut-off score is used as ≥14. To assess the robustness of this threshold, sensitivity analyses were conducted using alternative cut-off values of ≥13 and ≥15. For an ASRS-5 cut-off of ≥13, the group difference was not statistically significant (t = 1.39, *p* = 0.17), with a small-to-moderate effect size (Cohen’s d = 0.43) (pADHDt: *n* = 22, mean ± SD = 561.90 ± 775.03; control: *n* = 20, mean ± SD = 329.46 ± 109.18). Similarly, when a more stringent cut-off of ≥15 was applied, the group difference was not statistically significant (t = −0.70, *p* = 0.48), and the effect size was small (Cohen’s d = 0.23) (pADHDt: *n* = 15, mean ± SD = 384.55 ± 228.77; control: *n* = 27, mean ± SD = 488.25 ± 695.32). Overall, effect sizes across alternative cut-off values were consistent with the primary analysis. ASRS-5 mean values were the only statistically different value as it higher in group 1 than in group 2 (*p* < 0.05). Group 1 made significantly more total errors and showed significantly lower Go Correct RT and mean reaction time compared to Group 2 (*p* < 0.05). However, there were no significant differences between the groups in No-Go Wrong RT (*p* > 0.05) (Table 2).

**Table 1:** Comparison of participants’ descriptive features by groups (*N* = 42).

Variable	Groups		Test (*p*)
	Group 1	Group 2	
	*n =* 21	*n =* 21	
Age, (year)			*t =* −1.451 *p =* 0.155
X ± SS	22.76 ± 3.25	24.38 ± 3.94	
M (min-max)	22 (20–33)	24 (19–33)	
ASRS-5			***t =* 9.510** ***p <* 0.001**
X ± SS	16.95 ± 2.58	8.90 ± 2.90	
M (min-max)	16 (14–22)	9 (4–14)	
Gender, *n* (%)			*χ^2^ =* 0.889 *p =* 0.346
Male	7 (%33.3)	10 (%47.6)	
Female	14 (%66.7)	11 (%52.4)	
Body Mass Index, (kg/m^2^)			*t =* −0.999 *p =* 0.324
X ± SS	23.23 ± 4.91	24.61 ± 4.00	
M (min-max)	21.5 (18–35)	23.9 (19–34)	
Surgery history, *n* (%)			*χ^2^ =* 1.024 *p =* 0.311
No	20 (%95.2)	21 (%100)	
Yes	1 (%4.8)	0 (%0)	
Ear infection, *n* (%)			*χ^2^ =* 0.359 *p =* 0.549
No	20 (%95.2)	19 (%90.5)	
Yes	1 (%4.8)	2 (%9.5)	
Sensory sensitivity, *n* (%)			*χ^2^ =* 0.171 *p =* 0.679
No	17 (%81)	18 (%85.7)	
Yes	4 (%19)	3 (%14.3)	
Physical activity, *n* (%)			*χ^2^ =* 0.104 *p =* 0.747
No	14 (%66.7)	13 (%61.9)	
Yes	7 (%33.3)	8 (%38.1)	

Chi-Square Test (χ2); Dependent Sample t Test (t); the following are the descriptive statistics: mean (X), standard deviation (SD), median (M), minimum (min), maximum (max), number (n), and percentage (%). Statistical significance is indicated by bolded sections (*p* < 0.05).

**Table 2:** Comparison of (SART reports) attention measurements by groups (*N* = 42).

Variable	Groups		Test (*p*)
	Group 1	Group 2	
	*n =* 21	*n =* 21	
Total errors			***t =* 3.401** ***p =* 0.002**
X ± SS	9.00 ± 4.35	4.62 ± 3.99	
M (min-max)	10 (1–17)	3 (0–15)	
No-Go Wrong RT			*t =* −0.592 *p =* 0.557
X ± SS	113.30 ± 53.30	127.58 ± 96.77	
M (min-max)	110.1 (6–186)	93.4 (0–361)	
Go correct RT			***t =* −2.164** ***p =* 0.036**
X ± SS	184.39 ± 60.78	233.44 ± 84.20	
M (min-max)	164.6 (93–304)	235.7 (88–371)	
RT mean			***t =* −2.052** ***p =* 0.047**
X ± SS	240.89 ± 61.66	289.35 ± 88.93	
M (min-max)	223.9 (146–361)	292.9 (142–427)	

RT “stands for “Reaction Time.” The independent sample t test (t) is performed. Mean (X), standard deviation (SD), median (M), minimum (min), and maximum (max) values are provided as descriptive statistics. Statistical significance is indicated by the bolded sections (*p* < 0.05).

**Figure 3:**
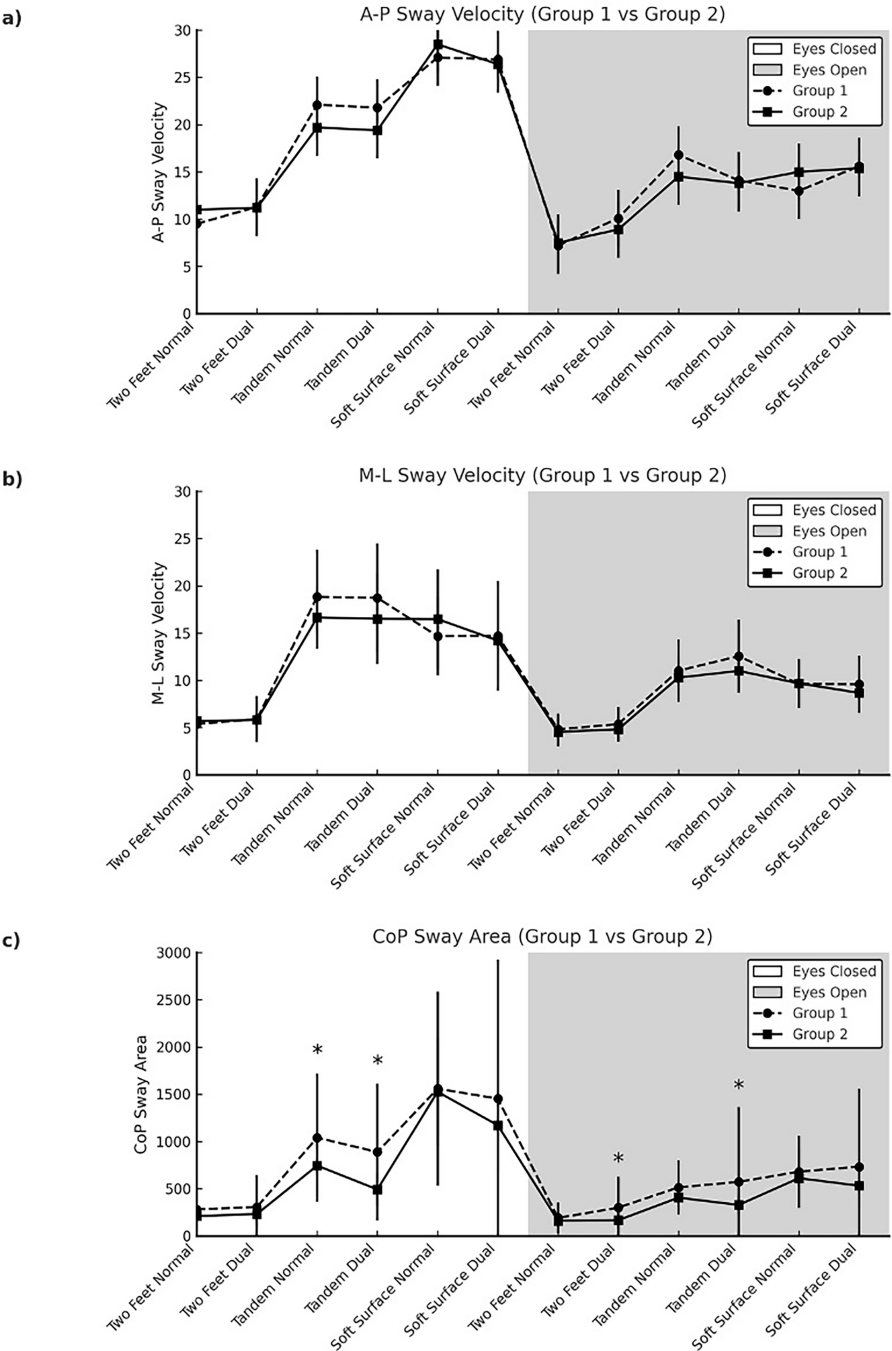
Graphical representation of changes in postural control variables: **(a)** A–P sway velocity, **(b)** M–L sway velocity, and **(c)** sway area among participants in Group 1 and Group 2 under different conditions.

Regarding postural control tests results, Table 3 shows A-P, M-L sway velocity and sway area mean changes in all tested conditions. No statistically significant difference in A-P and M-L sway velocity assessment in group 1 and group 2 (*p* > 0.05) (Figure 3).

**Table 3:** Comparisons of the intra-group and inter-group of A-P, M-L sway velocity and elliptical area.

Postural control parameters		Groups		Between-group comparison*P*, η2
		Group1		Group 2
		*n =* 21		*n =* 21
		X ± SD		X ± SD
A-P velocity (eyes closed)				
Two feet standing	Normal task	9.57 ± 2.87^C^	10.81 ± 2.68^C^	*p* = 0.156 η2 = 0.050
	Dual task	11.48 ± 5.07^C^	11.43 ± 3.03^C^	*p* = 0.971 η2 = 0.001
Tandem standding	Normal task	21.78 ± 7.08^B^	19.60 ± 4.90^B^	*p* = 0.254 η2 = 0.032
	Dual task	21.51 ± 9.09^B^	19.40 ± 6.36^B^	*p* = 0.389 η2 = 0.019
Standing on soft surface	Normal task	26.87 ± 7.78^A^	28.38 ± 6.95^A^	*p* = 0.511 η2 = 0.011
	Dual task	27.43 ± 9.16^A^	26.21 ± 6.01^A^	*p* = 0.612 η2 = 0.006
Test statistics ϕ		***p* < 0.001 η2 = 0.871**	***p* < 0.001 η2 = 0.844**	
A-P velocity (eyes open)				
Two feet standing	Normal task	6.92 ± 1.26^C^	7.43 ± 1.73^BC^	*p* = 0.283 η2 = 0.029
	Dual task	10.67 ± 6.59^B^	8.94 ± 2.12^B^	*p* = 0.259 η2 = 0.032
Tandem standding	Normal task	14.59 ± 4.24^A^	14.03 ± 2.71^A^	*p* = 0.616 η2 = 0.006
	Dual task	16.70 ± 8.87^A^	14.13 ± 2.65^A^	*p* = 0.211 η2 = 0.039
Standing on soft surface	Normal task	13.67 ± 2.62^A^	15.03 ± 5.89^A^	*p* = 0.338 η2 = 0.023
	Dual task	15.83 ± 6.31^A^	15.14 ± 4.05^A^	p = 0.679 η2 = 0.004
Test statistics ϕ		***p* < 0.001 η2 = 0.793**	***p* < 0.001 η2 = 0.763**	
M-L velocity (eyes closed)				
Two feet standing	Normal task	5.38 ± 1.72^D^	5.70 ± 1.98^D^	*p* = 0.582 η2 = 0.008
	Dual task	5.90 ± 2.42^D^	5.81 ± 1.97^D^	*p* = 0.890 η2 = 0.001
Tandem standding	Normal task	18.84 ± 4.94^A^	16.65 ± 3.31^AB^	*p* = 0.099 η2 = 0.066
	Dual task	18.73 ± 5.74^A^	16.51 ± 4.80^AB^	*p* = 0.181 η2 = 0.044
Standing on soft surface	Normal task	14.67 ± 4.14^BC^	16.48 ± 5.28^B^	*p* = 0.223 η2 = 0.037
	Dual task	14.71 ± 5.79^BC^	14.21 ± 4.34^C^	*p* = 0.749 η2 = 0.003
Test statistics ϕ		***p* < 0.001 η2 = 0.908**	***p* < 0.001 η2 = 0.874**	
M-L velocity (eyes open)				
Two feet standing	Normal task	4.81 ± 1.68^C^	4.54 ± 1.50^C^	*p* = 0.586 η2 = 0.007
	Dual task	5.37 ± 1.79^C^	4.81 ± 1.31^C^	p = 0.259 η2 = 0.032
Tandem standding	Normal task	11.02 ± 3.29^AB^	10.29 ± 2.29^AB^	*p* = 0.409 η2 = 0.017
	Dual task	12.56 ± 3.86^A^	11.00 ± 1.97^A^	*p* = 0.108 η2 = 0.063
Standing on soft surface	Normal task	9.63 ± 2.33^B^	9.68 ± 2.59^AB^	*p* = 0.950 η2 = 0.001
	Dual task	9.59 ± 3.00^B^	8.67 ± 2.12^B^	*p* = 0.257 η2 = 0.032
Test statistics ϕ		***p* < 0.001 η2 = 0.812**	***p* < 0.001 η2 = 0.787**	
Elliptical area (eyes closed)				
Two feet standing	Normal task	283.52 ± 228.44^D^	211.48 ± 105.46^D^	*p* = 0.197 η2 = 0.041
	Dual task	307.08 ± 338.14^D^	233.19 ± 137.19^D^	*p* = 0.359 η2 = 0.021
Tandem standding	Normal task	1041.22 ± 678.33^B^	745.49 ± 329.07^C^	**p = 0.040 η2 = 0.077**
	Dual task	888.81 ± 724.29^B^	493.19 ± 180.39^D^	**p = 0.020 η2 = 0.129**
Standing on soft surface	Normal task	1558.87 ± 1025.99^A^	1525.57 ± 570.20^A^	*p* = 0.897 η2 = 0.001
	Dual task	1453.79 ± 1470.97^AB^	1172.65 ± 482.69^BC^	*p* = 0.410 η2 = 0.017
Test statistics ϕ		***p* < 0.001 η2 = 0.706**	***p* < 0.001 η2 = 0.720**	
Elliptical area (eyes open)				
Two feet standing	Normal task	191.51 ± 163.32^D^	162.40 ± 77.93^D^	*p* = 0.465 η2 = 0.013
	Dual task	300.78 ± 327.60^C^	167.68 ± 82.10^D^	**p = 0.048 η2 = 0.075**
Tandem standding	Normal Task	515.02 ± 285.15^AB^	406.30 ± 140.83^BC^	*p* = 0.125 η2 = 0.058
	Dual task	573.06 ± 792.36^AB^	329.38 ± 106.42^CD^	**p = 0.047 η2 = 0.074**
Standing on soft surface	Normal task	681.49 ± 381.30^A^	612.57 ± 315.32^A^	*p* = 0.527 η2 = 0.010
	Dual task	734.49 ± 827.24^A^	532.51 ± 229.22^AB^	*p* = 0.287 η2 = 0.028
Test statistics ϕ		***p* < 0.001 η2 = 0.709**	***p* < 0.001 η2 = 0.617**	

Number of effects (η2), ϕ comparing different groups. The mean (X) and standard deviation (SD) are the two main pieces of descriptive statistics. The bolded parts show statistical significance (*p* < 0.05). If there are substantial differences between letters in the same row or column, then A > B > C > D (Bonferroni *p* < 0.05).

### 3.1 A-P sway velocity

In within-group comparisons under eyes-closed conditions, both groups exhibited significant differences in A-P sway velocity across the two-feet standing, tandem standing, and standing on a soft surface positions during both the normal and dual tasks (Group 1: *p* < 0.001, η^2^ = 0.871; Group 2: *p* < 0.001, η^2^ = 0.844). *Post hoc* comparisons revealed that, under eyes-closed conditions, the highest A-P sway velocity was observed in the standing on a soft surface position, followed by tandem standing and two-feet standing (*p* < 0.05) in both groups. Under eyes-open conditions, significant differences were also found within groups for the same postures and task conditions (Group 1: *p* < 0.001, η^2^ = 0.793; Group 2: *p* < 0.001, η^2^ = 0.763). *Post hoc* analyses indicated that A-P sway velocity was highest in the standing on a soft surface and tandem standing positions, and lowest in the two-feet standing position (*p* < 0.05). Additionally, within both groups, a significant difference between normal and dual tasks was observed only in the two-feet standing condition, where sway velocity was significantly lower during the dual task (*p* < 0.05) (Table 2).

### 3.2 M-L sway velocity

In within-group comparisons under eyes-closed conditions, both groups showed significant differences in M-L sway velocity across the two-feet standing, tandem standing, and standing on a soft surface positions for both the normal and dual tasks (Group 1: *p* < 0.001, η^2^ = 0.908; Group 2: *p* < 0.001, η^2^ = 0.874). *Post hoc* analyses revealed that M-L sway velocity was highest in the tandem standing position, followed by two-feet standing and standing on a soft surface (*p* < 0.05). Similarly, under eyes-open conditions, both groups demonstrated significant differences in sway velocity across the three standing conditions (group 1: *p* < 0.001, η^2^ = 0.812; Group 2: *p* < 0.001, η^2^ = 0.787). Post hoc comparisons showed that the highest M-L sway velocity occurred during tandem standing, followed by standing on a soft surface and two-feet standing. However, no significant differences were observed between normal and dual task conditions in the two-feet standing position (*p* > 0.05) (Table 2).

### 3.3 The CoP sway area

Table 3 shows the CoP sway area measurements in the eyes closed and eyes open conditions. On both normal and dual task situations with closed eyes, the CoP sway area was significantly higher in group 1 while they were in the Tandem position (*p* = 0.040 η2 = 0.077 and *p* = 0.020 η2 = 0.129, respectively). In the eyes-open condition, group 1 also showed significantly higher sway area in the two feet standing position under dual task (*p* = 0.048, η2 = 0.075) and in the Tandem stance under dual task (*p* = 0.047, η2 = 0.074). Other comparisons did not show significant group differences (*p* > 0.05). There were significant differences in the ellipse area across different postural surfaces and task conditions within each group (*p* < 0,001).

## 4 Discussion

This study aimed to examine the connections between postural control and sustained attention in individuals with pADHDt. Sensorimotor perturbations included altered proprioception via soft surface, modified visual input, and cognitively demanding dual-task conditions. The results showed that within-group changes in postural sway were similar in both groups. In both groups, A-P sway velocity was highest on soft surfaces, followed by tandem and upright stances. However, M-L sway velocity patterns differed, with the tandem stance producing the highest sway velocities. In the context of the CoP sway area, significant differences were found between the two groups under both eyes-open and eyes-closed conditions in dual-task tasks performed in a tandem stance. Furthermore, under open-eye conditions, there was a significant difference in the dual task during the two-feed posture.

In the SART task, which is used to assess participants’ ability to sustain attention, participants are expected to respond to certain stimuli while not responding to others. Individuals with pADHDt tend to make more commission errors (responding when they should not) and omission errors (failing to respond when they should). These patterns are commonly interpreted as behavioral indicators of inattention and impulsivity. Omission errors suggest difficulty maintaining attention. Previous studies have shown that adults with ADHD traits make significantly more errors on the SART, and that children and adolescents with ADHD exhibit higher omission error rates and greater reaction time variability in longitudinal assessments (Gau et al., 2022; Machida et al., 2022; Manly et al., 1999; Robertson et al., 1997; Thomson et al., 2020). However, not all studies have reported consistent results. For example, Chan (2001) found no significant differences in SART performance between ADHD and control groups. In this study, individuals with pADHDt performed significantly worse on the sustained attention task, as indicated by a higher number of total and omission errors, as well as longer reaction times. Furthermore, these results highlight that the pADHDt group differed from the control group in terms of attentional performance.

Notably, group comparisons of body sway velocity revealed no significant differences, aligning with findings by Jansen et al. (2019). However, much of the existing literature focuses on children, where increased sway velocity in ADHD populations is frequently reported (Bucci et al., 2016; Caldani et al., 2019; Shorer et al., 2012). In the study, the result of absence of group differences in sway velocity in adults may reflect developmental compensation of postural control mechanisms over time. As previous research has shown, postural control undergoes refinement during adolescence, particularly through improved sensory integration (Marta, 2017; Paschaleri et al., 2022). Thus, deficits observed in childhood may be mitigated in adulthood by maturation of sensorimotor systems.

Further within-group analyses revealed that, under eyes-open conditions, dual-tasking significantly increased CoP sway area in the pADHDt group during upright stance, whereas the control group exhibited reduced sway area under the same conditions—indicating improved postural stability. Under eyes-closed conditions, the control group again showed reduced sway area in both the soft surface and tandem stances during dual-tasking, whereas the pADHDt group showed no significant change. Dual-tasking improves performance in typically developing adults but does not benefit individuals with pADHDt.

Maintaining upright posture is not a purely automatic process; rather, it requires attentional resources. This demand for attention in regulating postural sway area becomes particularly evident when an individual performs a concurrent secondary task. The literature has shown that the direction of attention affects motor performance (Masters, 1992; Wulf et al., 2001). In this study, backward counting was used as a verbal dual task as a cognitive load. The verbal dual task used diverted attention away from the physical task being performed. The literature discusses two approaches to how the direction of attention and cognitive control affect performance. These are the Reinvestment Theory and the Constrained Action Hypothesis. Both approaches emphasize the negative effects of conscious attention and control on motor performance. Based on this information, it was expected that postural stability would be better in the verbal backward counting dual task because conscious attention was focused on the cognitive task. However, in the current study, it was observed that the dual task did not improve motor performance by diverting attention away from the motor task in individuals with the pADHDt. In other words, the pADHDt, participants had difficulty directing their attention outward. These observations align with prior theoretical frameworks, as suggested by Masters (1992), Wulf et al. (2001), and Seidler et al. (2010), which emphasize that while external attentional focus or dual-task conditions typically enhance automatic motor control, individuals with the pADHDt may struggle to effectively redirect their attention, limiting the expected performance benefits under such conditions.

Previous findings suggest that balance impairments observed in children with attention difficulties may be compensated for in adulthood due to improved sensory integration and postural control development. The results obtained after the dual task here suggest that it is caused by problems in directing attention in dual tasks rather than the sensory system. One possible explanation is that individuals with pADHDt may have limited cognitive resources or impaired switching between cognitive and motor domains, making them less able to benefit from dual-task facilitation. With the problem in orienting attention, postural control cannot become automatic, and thus postural control performance decreases.

It should also be noted that the differences observed in postural control variables in the present study do not indicate a balance problem. The pADHDt group remained within the limits of stability. The findings obtained reflect a difference rather than a deficit. In addition, this behavior observed in individuals with pADHDt may also be defined as an exploratory behavior. With task learning, the difference between groups may no longer be present. Further studies are needed to determine these uncertainties.

Among CoP-derived parameters, sway velocity and sway area are the most commonly used and validated indicators of postural stability. Sway velocity, which reflects the net neuromuscular effort required for maintaining balance, is generally considered a highly sensitive and reliable metric (Paillard and Noé, 2015; Liang et al., 2021). Sway area, on the other hand, refers to the total area covered by the CoP trajectory in the A-P and M-L directions, with smaller areas indicating better postural performance (Liang et al., 2021; Tongar and Yazici-Mutlu, 2024). This raises an important question: what do discrepancies between postural control variables actually reflect? And what do they tell us about the underlying control processes? However, the literature does not establish a strict pattern regarding how these two parameters respond across different conditions or populations. We suggest that, particularly in healthy young adults, postural control may involve dynamic and transient internal calibrations—a kind of exploratory search within stability boundaries. This process may manifest as changes in sway velocity under visual perturbations, while task-driven attentional demands may highlight performance. A similar interpretation was proposed by Liang et al. (2021).

This study has several limitations. Participants were classified using a self-report tool rather than a clinical diagnosis, which may have introduced variability. Because participants were not clinically diagnosed, the present findings should not be generalized to individuals with ADHD but rather to non-clinical populations exhibiting elevated ADHD-related attentional traits. The lack of an objective cognitive cut-off score as an inclusion criterion may be considered a limitation, potentially increasing attentional performance variability across participants. The cognitive load involved only one dual-task (verbal backward counting), whereas other task types might yield different effects. Additionally, limited research on adults with pADHDt makes it difficult to fully contextualize the findings, highlighting the need for further studies in this population.

## 5 Conclusion

In conclusion, adults with pADHDt did not exhibit postural performance facilitation under dual task conditions. Unlike controls, they did not demonstrate from dual-task facilitation, potentially due to attentional allocation difficulties. Given the non-clinical and cross-sectional nature of the present study, these findings should be interpreted as reflecting trait-level attentional variability rather than clinically diagnosed ADHD. While no direct clinical recommendations can be drawn, the results highlight the importance of considering attentional demands in balance assessment paradigms and support further investigation of cognitive–motor interference and sensory integration mechanisms. These findings highlight the importance of considering attentional demands in balance assessments and suggest further investigation into sensory integration and cognitive-motor interference in this population.

## Data Availability

The raw data supporting the conclusions of this article will be made available by the authors, without undue reservation.
